# Propofol Inhibits Lipopolysaccharide-Induced Tumor Necrosis Factor-Alpha Expression and Myocardial Depression through Decreasing the Generation of Superoxide Anion in Cardiomyocytes

**DOI:** 10.1155/2014/157376

**Published:** 2014-08-11

**Authors:** Jing Tang, Ji-Jie Hu, Chun-Hua Lu, Jia-Ni Liang, Jin-Fang Xiao, You-Tan Liu, Chun-Shui Lin, Zai-Sheng Qin

**Affiliations:** ^1^Department of Anesthesiology, Nanfang Hospital, Southern Medical University, Guangzhou, Guangdong 510515, China; ^2^Department of Orthopedics and Traumatology, Nanfang Hospital, Southern Medical University, Guangzhou, Guangdong 510515, China; ^3^Department of Anesthesiology, The University of Hongkong Shenzhen Hospital, Shenzhen, Guangdong 518053, China

## Abstract

TNF-*α* has been shown to be a major factor responsible for myocardial depression in sepsis. The aim of this study was to investigate the effect of an anesthetic, propofol, on TNF-*α* expression in cardiomyocytes treated with LPS both *in vivo* and *in vitro*. In cultured cardiomyocytes, compared with control group, propofol significantly reduced protein expression of gp91phox and phosphorylation of extracellular regulated protein kinases 1/2 (ERK1/2) and p38 MAPK, which associates with reduced TNF-*α* production. In *in vivo* mice studies, propofol significantly improved myocardial depression and increased survival rate of mice after LPS treatment or during endotoxemia, which associates with reduced myocardial TNF-*α* production, gp91phox, ERK1/2, and p38 MAPK. It is concluded that propofol abrogates LPS-induced TNF-*α* production and alleviates cardiac depression through gp91phox/ERK1/2 or p38 MAPK signal pathway. These findings have great clinical importance in the application of propofol for patients enduring sepsis.

## 1. Introduction

Endotoxemia or sepsis is a major consequence of infectious diseases, which causes multiple organ injury, including injury of the cardiovascular system, and becomes one of the leading causes of death in patients in the intensive care unit (ICU) [1, 2]. It has been demonstrated that cardiomyocytes are the major local source of TNF-*α*, a proinflammatory cytokine, in the myocardium during sepsis and TNF-*α* is responsible for LPS induced cardiac function [3] and for myocardial depression induced by endotoxemia [4, 5]. Nicotinamide adenine dinucleotide phosphate oxidase (NADPH oxidase) is an enzyme system that catalyses the NADPH-dependent reduction of oxygen to superoxide anion (O_2_^−^) and consists of multisubunits including Nox2 (gp91phox), p22phox, p40phox, p47phox, p67phox, and Rac [6]. Previous studies have proven that gp91phox-containing NADPH oxidase activity plays a pivotal role in LPS-induced TNF-*α* expression in the heart. This signaling pathway involves O_2_^−^ generation and activation of ERK1/2 and p38 MAPK. Moreover, gp91phox-containing NADPH oxidase contributes to myocardial dysfunction during endotoxemia [7].

Propofol is one of the most widely used in anesthesia induction and maintenance, as well as sedation for ICU patients. Patients with peritonitis, infection, or sepsis may sometimes receive propofol for sedation [8–10]. Propofol acts through multiple mechanisms that may influence the pathophysiological process of endotoxemia. It has been proven that propofol can protect the cell membrane from lipid peroxidation by inhibiting the production of malondialdehyde [11]. Antioxidant effect of propofol resembles that of vitamin E. Therefore, due to its anti-inflammatory and antioxidant properties, propofol can decrease the production of proinflammatory cytokines, such as TNF-*α*, inducible nitric oxide synthase (iNOS), and interleukins (IL)-1, -6, -8, and -10. It can also inhibit neutrophil chemotaxis, attachment, and migration, phagocytosis, decrease the generation of reactive oxygen species, scavenge free radicals, and inhibit platelet aggregation [12–14]. However, the exact molecular mechanism of how propofol exerts anti-inflammatory or antioxidant properties such as inhibiting the release of TNF-*α* is still unclear.

Recently, studies demonstrated that, in endotoxemia or sepsis, propofol administration is associated with cardiovascular protective effects, including decreased blood pressure and systemic vascular resistance [15]. Cardiovascular dysfunction is one of the leading causes of death in septic shock patients, and the local source of TNF-*α* in the myocardium plays a critical role in cardiac failure during sepsis. Since gp91phox-containing NADPH oxidase activity and the subsequent generation of O_2_^−^ are mainly responsible for the production of TNF-*α* in cardiomyocytes [7], we hypothesized that propofol could inhibit TNF-*α* expression and O_2_^−^ production in cardiomyocytes and improve myocardial depression. To prove this hypothesis, neonatal cardiomyocytes from C57BL/6 mice were used and we found that propofol could decrease the generation of TNF-*α* in cardiomyocytes and alleviate cardiac failure through its inhibitory effect on the production of O_2_^−^ during sepsis.

## 2. Material and Methods

### 2.1. Materials

Propofol was prepared from Diprivan (Zeneca Limited, Macclesfield, Cheshire, UK). Intralipid was prepared from Sino-Swed Pharmaceutical Corp, Ltd (Jiangsu, China). Lipopolysaccharides (*Salmonella typhosa*), *β*-NADH, lucigenin, DPI, PD98059, and SB203580 were purchased from Sigma-Aldrich (St. Louis, MO, USA). Liberase TH was obtained from Roche (Mannheim, Germany). Mouse TNF-*α* ELISA kit was purchased from eBioscience (San Diego, CA, USA). Quantitative real-time PCR mix buffer was obtained from Promega (Madison, WI, USA). MTT, dimethyl sulfoxide (DMSO), TRIzol, and all culture medium and supplements were purchased from Invitrogen (Carlsbad, CA, USA).

### 2.2. Animals

Experimental protocols were approved by the local council of ethics and performed in accordance with the Guidelines for the Care and Use of Laboratory Animals of Nanfang Hospital. In accordance with the guidelines of the International Association for the Study of Pain as published in Pain 1983, 16: 109-110, after all the operations were done, animals were anesthetized with urethane, so animals did not feel pain or discomfort during the experiments and the minimum possible pain or stress had been imposed on the animals. Eight-week-old C57BL/6 mice adopted from the experimental animal center of Southern Medical University with a mean body weight of 25 g were randomly assigned into four groups. (1) Control group received intraperitoneal (i.p.) injections of 10% intralipid; (2) propofol alone group received propofol (50 mg/kg, i.p.); (3) LPS group received 10% intralipid pretreatment followed by LPS (5 mg/kg, i.p.); (4) LPS + propofol group received propofol (50 mg/kg, i.p.) pretreatment followed by LPS (5 mg/kg, i.p.).

Survival was monitored in mice in the following four groups of mice: (1) control group received intraperitoneal (i.p.) injections of 10% intralipid; (2) propofol alone group received propofol (50 mg/kg, i.p.); (3) LPS group received 10% intralipid pretreatment followed by LPS (20 mg/kg, i.p.); (4) LPS + propofol group received propofol (50 mg/kg, i.p.) pretreatment followed by LPS (20 mg/kg, i.p.).

### 2.3. Preparation of Neonatal Mouse Cardiomyocytes

Neonatal hearts from C57BL6 mice born within 24 h were minced in a nominally Ca^2+^ and Mg^2+^ free D-Hanks balance solution. Cardiac myocytes were dispersed by the addition of Liberase TH with a final concentration of 22.5 *μ*g/mL in D-Hanks solution and incubated in 37°C water bath for 10 min. After being mixed by pipette for about 2–4 min, the supernatant was collected into a 15 mL clean tube. Then new fresh digestion buffer was added for another round of incubation in 37°C water bath. The cell suspension was centrifuged at 1200 rpm for 5 min to obtain a cell pellet and the debris of heart was redigested one more time by Liberase TH and collected in the same tube. Cells were then suspended in M199 medium supplemented with 10% fetal bovine serum (FBS) and 1% penicillin-streptomycin solution (PS) and preplated for 60 min to remove noncardiomyocytes. Then the cell suspension was filtered through a polypropylene macroinvolved porous filter (mesh opening 105 *μ*m, Spectra/Mesh, Spectrum Medical Industries). The cardiomyocytes were plated at a density of 5 × 10^5^ cells/mL in M199 supplemented with 10% FBS and 1% PS on 24-well plates precoated with 1% gelatin. Cells were incubated at 37°C in a humidified atmosphere containing 5% CO_2_. A confluent monolayer of spontaneously beating cells was formed within 2 days and the purity of neonatal mouse cardiomyocytes was about 98% according to anti-troponin immunostaining.

### 2.4. Cell Viability

The MTT assay was used to measure cell viability. After intralipid or propofol treatment, neonatal cardiomyocytes were washed twice with Krebs-HEPES buffer (115 mmol/L NaCl, 24 mmol/L NaHCO_3_, 5 mmol/L KCl, 1 mmol/L MgCl_2_, 2.5 mmol/L CaCl_2_, 25 mmol/L HEPES, and 1% BSA, pH 7.4) without glucose and incubated in 1 mL Krebs-HEPES buffer with 0.5 mg/mL MTT for 60 min at 37°C. The supernatant was discarded, and cells in each well were lysed with 500 *μ*L of 2-propanol and incubated for 1 h at room temperature. The supernatant was removed and 150 *μ*L DMSO was added. The samples were read at 490 nm in a spectrometer, measuring the cell viability: viability = OD treatment/OD control × 100%.

### 2.5. Western Blot Analysis

Total proteins were extracted from the neonatal cardiomyocytes or cardiac tissues with the lysis buffer (20 mM Tris pH 7.5, 150 mM NaCl, 1.0 mM EDTA, 1.0 mM EGTA, 0.1% Triton X-100, 2.5 mM sodium pyrophosphate, and 1.0 mM *β*-pyrophosphate glycerol) supplemented with 1.0 mM Na_3_VO_4_, 1 mM MPMSF, and a protease inhibitor cocktail. Protein in the supernatant was quantified using a micro-BCA protein assay kit (Pierce, Rockford, IL). A total of 40 *μ*g of protein in each sample was subjected to SDS-PAGE with 10% gels, followed by electrotransfer to polyvinylidene difluoride (PVDF) membranes. For gp91phox protein detecting, the membrane was probed with anti-gp91phox rabbit polyclonal antibody (1 : 1000; Abcam). For phospho/total p38MAPK and phospho/total ERK detecting, the membrane was probed with anti-phospho-p38MAPK (Thr180/Tyr182) rabbit polyclonal antibody (1 : 1000; Cell Signaling Technology), or anti-p38MAPK rabbit polyclonal antibody (1 : 1000; Cell Signaling Technology), or anti-phospho-ERK1/2 (Thr202/Tyr204) rabbit polyclonal antibody (1 : 1000; Cell Signaling Technology), or anti-ERK1/2 rabbit polyclonal antibody (1 : 1000; Cell Signaling Technology). Horseradish peroxidase-conjugated anti-rabbit immunoglobulin G (1 : 2000; Cell Signaling Technology) was used as secondary antibody. The membranes were examined with a Kodak image station 2000R apparatus (Kodak, Rochester, NY, USA). *β*-Actin was used as the control for equal loading of the protein.

### 2.6. Measurement of TNF-*α* Protein

TNF-*α* protein levels in the medium of neonatal cardiomyocytes or left ventricle (LV) myocardium were determined using a mouse TNF-*α* enzyme-linked immunosorbent assay (ELISA) kit (eBioscience, San Diego, USA) according to the manufacturer's instructions. 50 *μ*L of culture medium was used for TNF-*α* measurement. The LV tissues were homogenized in phosphate-buffered saline (PBS). After centrifugation, the supernatant was collected for protein concentrations. 200 *μ*g total protein was used for TNF-*α* measurement.

### 2.7. Analysis of TNF-*α* and gp91phox by Reverse Transcriptase-Polymerase Chain Reaction

Total RNA was extracted from cardiomyocytes or left ventricle myocardium with the TriZol reagent (Gibco-BRL) according to the manufacturer's instructions. TNF-*α* and gp91phox were amplified using real-time reverse transcription-polymerase chain reaction (RT-PCR) with gene-specific primers: gp91phox (GenBank database ID: U43384), 5′-ACGCCCTTTGCCTCCATTCT-3′ (sense), and 5′- GCTTCAGGGCCACACAGGAA-3′ (antisense); TNF-*α* (GenBank database ID: M13049), 5′-CCG ATG GGT TGT ACC TTG TC-3′ (sense), and 5′-GGG CTG GGT AGA GAA TGG AT-3′ (antisense), respectively.

### 2.8. Detection of Superoxide Anion by Lucigenin Chemiluminescence

NADH-dependent O_2_^−^ generation was measured in cell lysates by lucigenin-enhanced chemiluminescence (40 *μ*g of protein, 100 *μ*mol/L *β*-NADH, 5 *μ*mol/L lucigenin). The chemiluminescence was detected by a multilabel counter (SpectraMax M5; Molecular Devices, Sunnyvale, CA, USA). Replicates were incubated in the presence of the flavoprotein inhibitor (DPI, 10 *μ*M) to ensure that O_2_^−^ was generated from NADPH oxidase. The light signal was monitored for 3 sec and counts per second were presented as NADPH oxidase activity that was inhibitable by DPI.

### 2.9. *In Vivo* Cardiac Hemodynamics

Adult male C57BL/6 mice were randomly divided into four groups. (1) Control group received intraperitoneal (i.p.) injections of 10% intralipid; (2) propofol alone group received propofol (50 mg/kg, i.p.); (3) LPS group received 10% intralipid pretreatment followed by LPS (5 mg/kg, i.p.); (4) LPS + propofol group received propofol (50 mg/kg, i.p.) pretreatment followed by LPS (5 mg/kg, i.p.). After 6 h, mice were anesthetized by inhalation of 1% isoflurane and 99% oxygen. The mice were then ventilated at 120 strokes/minute with a rodent ventilator (Harvard Instruments, Holliston, MA) and kept on a warm pat to stabilize the body temperature at 37°C. A 1.4-F high-fidelity micromanometer catheter (Millar Instruments, Houston, TX) was inserted into the LV through carotid artery under continuous hemodynamic monitoring to measure LV pressure, volume, and heart rate. Hemodynamic parameters were analyzed with PVAN3.3 software (Millar Instruments).

### 2.10. Statistical Analysis

Quantitative data are expressed as the mean ± S.D. from at least three independent experiments. Statistical analysis was carried out using one-way ANOVA analysis and Newman-Keuls post hoc analysis. Survival studies were analyzed by Chi-square test. A *P* value of 0.05 or less was considered significant. Graphs and figures were made with Graphpad Prism 5.01 (GraphPad software, CA, USA).

## 3. Results

### 3.1. Toxicity of Propofol

**Figure 1:**
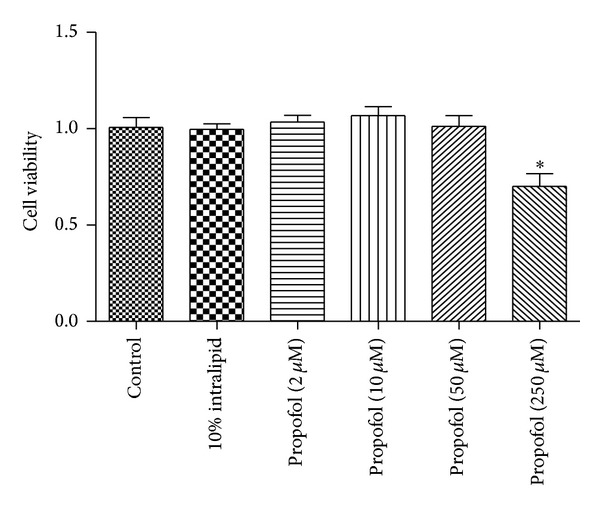
Effects of propofol treatment on cell viability of neonatal cardiomyocytes. Cultured neonatal cardiomyocytes seeded in 96-well plate were exposed to propofol at indicated concentrations for 24 h. The cell viability was determined by MTT assay. Data are representatives of three independent experiments with similar tendency. *P < 0.05 (*n* = 3), compared with control.

Since propofol is dissolved in 10% intralipid, cell viability was assayed by 3-(4,5-dimethylthiazol-2-yl)-2,5-diphenyltetrazolium bromide (MTT) assay to evaluate the toxicity of propofol and intralipid to isolated neonatal cardiomyocytes from C57BL/6 mice. Neonatal cardiomyocytes treated with 2, 10, and 50 *μ*M propofol for 24 h did not affect cell viability. However, when the concentration reached 250 *μ*M, propofol caused a significant decrease in cell viability (*P* = 0.021 versus control). Exposure of cultured neonatal cardiomyocytes to 10% intralipid did not influence cellular viability (Figure 1). We thus chose 50 *μ*M for the subsequent experiments with propofol pretreatment.

### 3.2. Propofol Inhibits the Upregulation of NADPH Oxidase in Cardiomyocytes Induced by LPS

**Figure 2:**
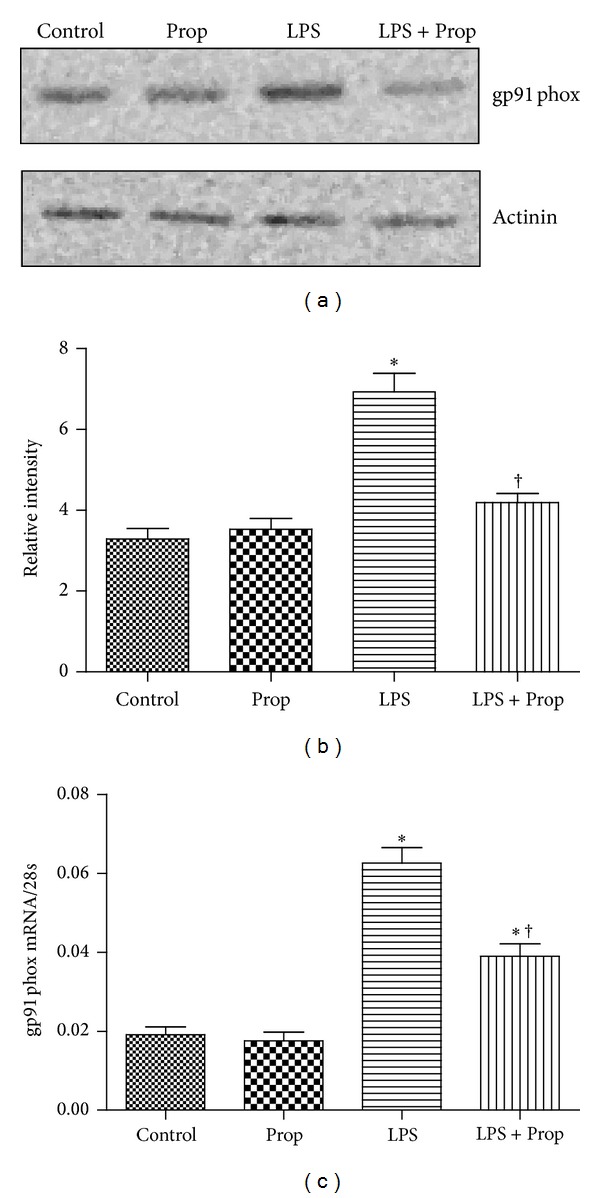
The inhibitory effect of propofol on gp91phox protein and mRNA expression in LPS-stimulated cardiomyocytes. Cardiomyocytes were pretreated with 10% intralipid or propofol (50 *μ*M) followed by LPS (4 *μ*g/mL) for 2 or 4 hours. (a) gp91phox protein was determined by western blot analysis at 4 hours after LPS treatment. (b) Gray intensity analysis of the western blot results of four groups. (c) Real time RT-PCR amplification for gp91phox mRNA (each bar represents the mean ± S.D, *P < 0.05, compared with control group; ^†^ *P* < 0.05, compared with LPS group; *n* = 4).

LPS increases gp91phox-containing NADH oxidase activity which is mainly responsible for the release of TNF-*α* in cardiomyocytes. As propofol is well known for its antioxidant activities, we measured the effect of propofol on the expression of gp91phox-containing NADH oxidase during LPS treatment in cardiomyocytes [7]. Cultured neonatal cardiomyocytes were divided into four groups: control, propofol alone, LPS, and LPS + propofol groups. As shown in Figure 2, LPS treatment increased gp91phox expression both in mRNA and protein level, which were abolished by propofol treatment, while propofol alone had no effect on gp91phox mRNA and protein expression compared with control group (Figure 2).

### 3.3. Effect of Propofol on the Generation of O_2_^−^ and TNF-*α* in Cardiomyocytes during LPS Treatment

**Figure 3:**
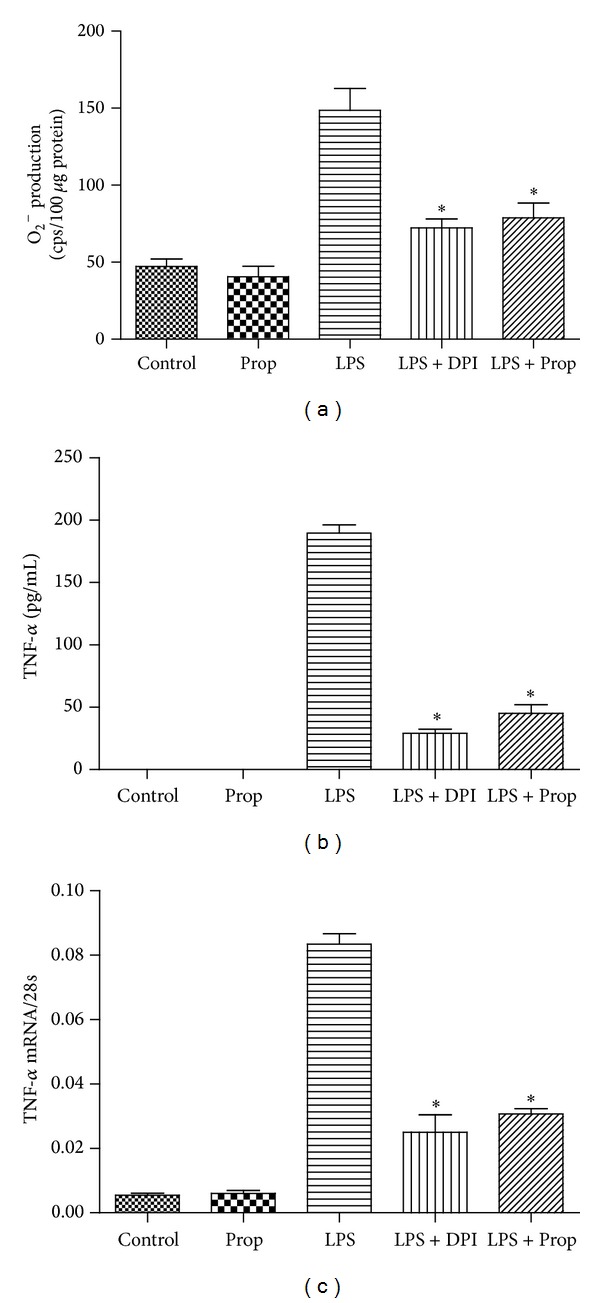
The inhibitory effect of propofol on O_2_^−^ generation and TNF-*α* expression in LPS-stimulated cardiomyocytes. Cardiomyocytes were pretreated with vehicle, DPI (50 *μ*M), or propofol (50 *μ*M) followed by LPS (4 *μ*g/mL) for 2 or 4 hours. (a) O_2_^−^ generation was determined by lucigenin-enhanced chemiluminescence at 2 hours after LPS treatment; (b) TNF-*α* protein measurement by ELISA at 4 hours after LPS treatment; (c) TNF-*α* mRNA measurement by real-time RT-PCR at 2 hours after LPS treatment (each bar represents the mean ± S.D, *P < 0.05, compared with LPS group; *n* = 4).

It has been proven that upregulation of NADPH oxidase expression induced by LPS is associated with an increase in O_2_^−^ generation [16]. Therefore, we measured the effect of propofol on the production of O_2_^−^ in cardiomyocytes treated with LPS. Compared with control or propofol alone groups, LPS dramatically increased O_2_^−^ generation and this effect could be inhibited by both propofol and NADH oxidase pharmacological inhibitors, diphenyleneiodonium (DPI) (Figure 3(a)). Since O_2_^−^ is mainly responsible for the production of TNF-*α* in cardiomyocytes during LPS treatment, we examined the role of propofol and DPI in TNF-*α* generation in cultured cardiomyocytes treated with LPS. As we expected, with the increase of O_2_^−^ generation, TNF-*α* expression was dramatically upregulated both in protein and mRNA levels after LPS treatment, while DPI and propofol decreased O_2_^−^ production and TNF-*α* expression both in protein and mRNA levels (Figures 3(b) and 3(c)). These results indicated that propofol could inhibit TNF-*α* expression both in protein and mRNA levels through downregulating NADH oxidase expression and NADH oxidase activity in cardiomyocytes.

### 3.4. Role of Propofol on O_2_^−^ Induced Phosphorylation of p38 and ERK1/2 in Cardiomyocytes after LPS Treatment

**Figure 4:**
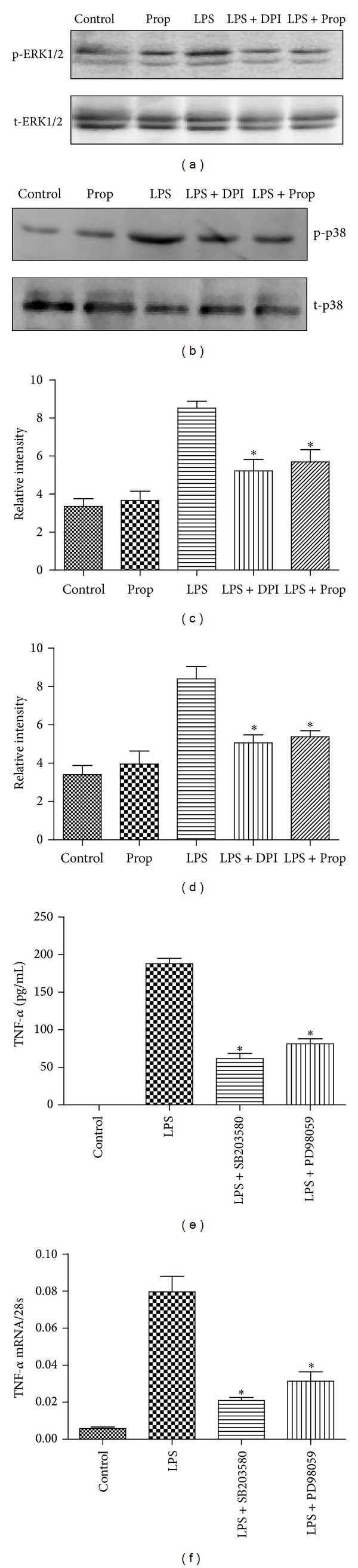
The inhibitory effect of propofol on ERK1/2 and p38 phosphorylation in LPS-stimulated cardiomyocytes. Cardiomyocytes were pretreated with vehicle, DPI (50 *μ*M), or propofol (50 *μ*M) followed by LPS (4 *μ*g/mL) for 2 hours. (a) The effect of propofol or DPI on ERK1/2 phosphorylation. (b) The effect of propofol or DPI on p38 phosphorylation. (c) Gray intensity analysis of the western blot results of ERK1/2 phosphorylation. (d) Gray intensity analysis of the western blot results of p38 phosphorylation. Cardiomyocytes were pretreated with vehicle, PD98059 (20 *μ*M), or SB203580 (10 *μ*M) followed by LPS (4 *μ*g/mL) for 4 hours. (e) TNF-*α* protein measurement by ELISA at 4 hours after LPS treatment; (f) TNF-*α* mRNA measurement by real time RT-PCR at 2 hours after LPS treatment (each bar represents the mean ± S.D, *P < 0.05, compared with LPS group; *n* = 4).

Although some studies have reported that propofol could inhibit the phosphorylation of p38 and ERK1/2, the molecular mechanism where propofol exerts this effect remained unknown. In the current study, we found that, compared with control and propofol alone groups, the increased O_2_^−^ generation induced by LPS increased the phosphorylation of p38 MAPK and ERK1/2. Inhibition of the activity of NADH oxidase with DPI or propofol downregulated LPS-induced increased O_2_^−^ generation, which associated with decreased phosphorylation levels of p38 MAPK and ERK1/2 (Figures 4(a)–4(d)). These results indicated that propofol downregulated LPS-induced p38 MAPK and ERK1/2 phosphorylation through inhibiting NADH oxidase expression and NADPH oxidase activity in cardiomyocytes. To further investigate the role of p38 MAPK and ERK1/2 activation in LPS-induced TNF-*α* expression, cardiomyocytes were treated with p38 MAPK signaling inhibitor, SB203580, and/or ERK1/2 signaling inhibitor, PD98059. We found that both SB203580 and PD98059 abrogated TNF-*α* mRNA and protein expression in LPS-treated cardiomyocytes (Figures 4(e) and 4(f)). These results suggested that propofol decreased the production of TNF-*α* through inhibiting O_2_^−^ induced phosphorylation of p38 MAPK and ERK1/2 in cardiomyocytes after LPS treatment.

### 3.5. The Effect of Propofol on the Production of TNF-*α* in the Myocardium of C57BL/6 Mice

**Figure 5:**
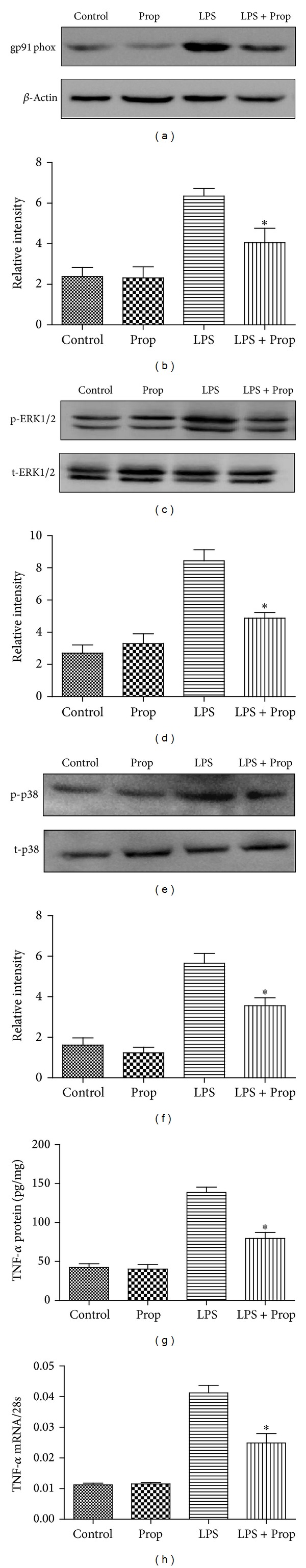
The inhibitory effect of propofol on NADPH oxidase/TNF-*α* signal pathway in LPS-stimulated myocardium of wild type C57BL/6 mice. Wild type C57BL/6 mice were divided into four groups: control group, propofol alone group, LPS group, and LPS + propofol group. (a) The effect of propofol on gp91phox protein expression in myocardium 2 hours after LPS treatment. (b) Gray intensity analysis of the western blot results of gp91phox expression. (c) The effect of propofol on ERK1/2 phosphorylation in myocardium 1 hour after LPS treatment. (d) Gray intensity analysis of the western blot results of ERK1/2 phosphorylation. (e) The effect of propofol on p38 phosphorylation in myocardium 1 hour after LPS treatment. (f) Gray intensity analysis of the western blot results of p38 phosphorylation. (g) TNF-*α* protein measurement in myocardium by ELISA at 4 hours after LPS treatment; (h) TNF-*α* mRNA measurement in myocardium by real time RT-PCR at 2 hours after LPS treatment (each bar represents the mean ± S.D, *P < 0.05, compared with LPS group; *n* = 5).

In cultured neonatal cardiomyocytes, our results showed that propofol could inhibit NADPH oxidase expression and NADH oxidase activity to downregulate the phosphorylation of p38 MAPK and ERK1/2 and ultimately to decrease LPS-induced TNF-*α* expression. To replicate these results in the adult myocardium, adult male C57BL/6 mice were divided into four groups: control, propofol alone, LPS, and LPS + propofol group. As shown in Figure 5, compared with control and propofol alone group, LPS significantly increased protein expression of gp91phox-containing NADPH oxidase in the myocardium, and this effect could be partially inhibited by propofol (Figures 5(a) and 5(b)). Similarly, propofol significantly reduced LPS-induced enhanced phosphorylation of p38 MAPK and ERK1/2 protein expression in the cardiac tissue of four groups (Figures 5(c)–5(f)). Finally, we measured TNF-*α* mRNA and protein expression in the myocardium of four groups and found that propofol treatment partially alleviated LPS-induced increased TNF-*α* expression both in mRNA and protein levels (Figures 5(g) and 5(h)).

### 3.6. Effect of Propofol on Myocardial Dysfunction during Endotoxemia

**Figure 6:**
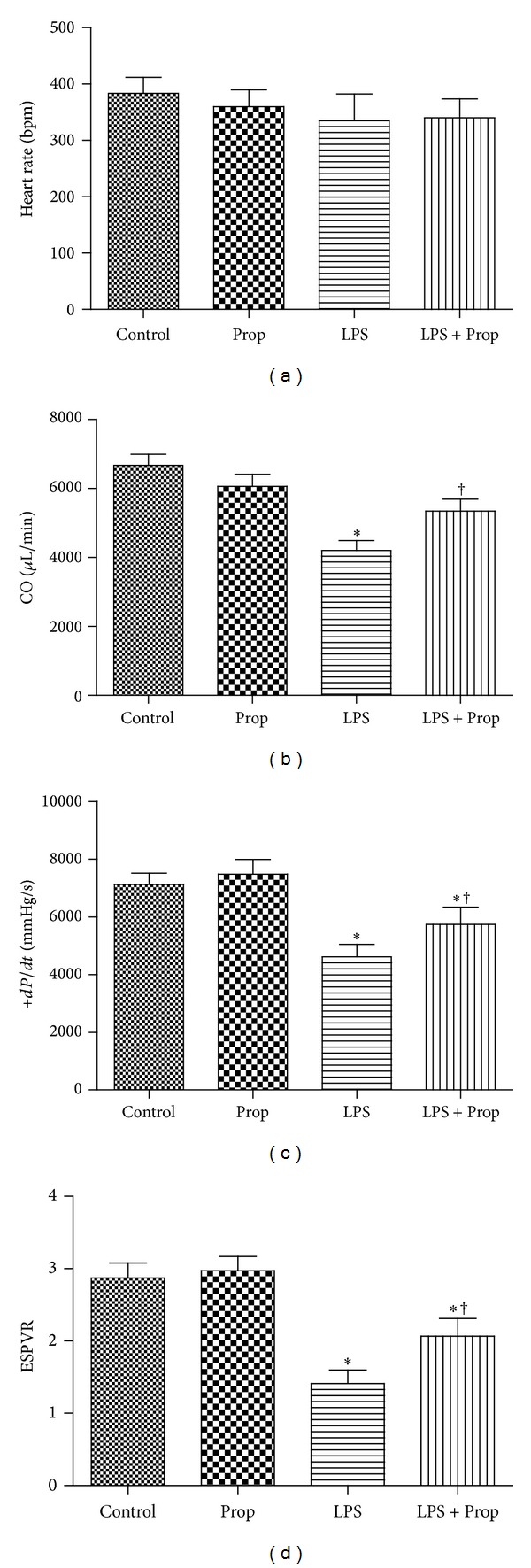
*In vivo* cardiac function during endotoxemia.Wild type C57BL/6 mice were divided into four groups: control group, propofol alone group, LPS group, and LPS + propofol group. After 6 h, cardiac function was determined using a Millar pressure-conductance catheter. Changes in LV, (a); heart rate, (b); CO, (c); +dp/dt, (d) ESPVR were presented (each bar represents the mean ± S.D, *P < 0.05 compared with control group; ^†^ *P* < 0.05 compared with LPS group, *n* = 6).

To explore whether or not propofol could improve myocardial depression induced by endotoxemia, C57BL/6 mice were treated with vehicle, propofol, LPS, or propofol + LPS separately. After 6 hrs of LPS* in vivo* treatment, cardiac function was determined. Although there was no change in heart rate, rate of contraction (+dp/dt), cardiac output (CO), and end systolic pressure volume relationship (ESPVR) were significantly reduced in endotoxemic mice compared with control and propofol alone animals indicating myocardial depression. Propofol, however, partially restored +dp/dt, CO, and ESPVR without affecting heart rate in endotoxemic mice (Figure 6). These results demonstrated that propofol alleviate myocardial dysfunction of endotoxemia in mice.

### 3.7. Effects of Propofol on the Survival Rate of Wild Type C57BL/6 Mice during Endotoxemia

**Figure 7:**
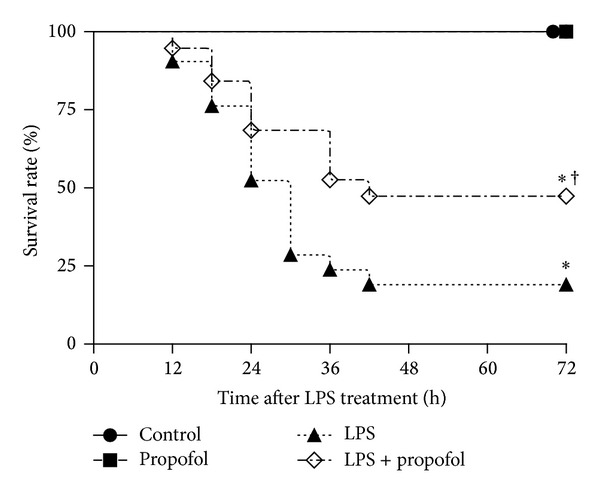
Effects of propofol on survival of mice treated with LPS. Wild type C57BL/6 mice were pretreated with 10% intralipid or propofol (50 mg/kg, i.p.) followed by LPS (20 mg/kg, i.p.) as described in Section 2. After LPS administration, survival of mice was monitored at every 12 h for 72 h. Survival was significantly decreased in LPS group (*n* = 16) and propofol partially reversed by propofol (*n* = 16) compared with control (*n* = 6) and propofol alone (*n* = 6) group (*P < 0.05 compared with control group; ^†^ *P* < 0.05 compared with LPS group).

To further evaluate the protective effect of propofol in mice with endotoxemia, the survival rate of control, propofol alone, LPS, and LPS + propofol groups was monitored. We found that mice injected with LPS were associated with a 72 h survival rate of about 20%. In contrast, LPS-injected mice treated with propofol had a higher survival rate of 46% at 72 h. Propofol significantly (*P* = 0.0375) increased the survival rate of animals with LPS injection. In the control and propofol alone group, no mice died within 72 h (Figure 7). These data indicated that treatment with propofol could significantly protect mice with endotoxemia from death.

## 4. Discussion

In the present study, we demonstrate that (i) propofol inhibits LPS-induced increased TNF-*α* production in cardiomyocytes, (ii) the inhibitory effect of propofol on TNF-*α* production in cardiomyocytes relies on its ability of suppressing the activity of NADH oxidase to decrease the generation of O_2_^−^ and phosphorylation of p38 MAPK and ERK1/2, (iii) propofol alleviates myocardial dysfunction in mice with endotoxemia, and (iv) propofol protects mice with endotoxemia from death. Thus, the protective and anti-inflammatory effects of propofol on cardiac dysfunction may contribute to the increased survival of mice with endotoxic shock.

Propofol (2,6-diisopropylphenol) is the most routinely used intravenous anesthetic for short-term sedation in surgery as well as in combined treatments for patients with critical illnesses [17, 18]. In addition to its anesthetic properties, propofol also exhibits anti-inflammation and antioxidant effects. In endotoxemia-induced septic model, propofol inhibits neutrophil functions, including chemotaxis, attachment, migration, and phagocytosis [19]. Propofol also suppresses proinflammatory cytokine production and inducible NO synthase/NO biosynthesis in endotoxin LPS-activated macrophages and peripheral blood mononuclear cells [20]. Propofol confers antioxidant activity by scavenging free radicals and peroxynitrite to decrease oxidative stress-induced lipid peroxidation [21]. In endotoxemia or sepsis, myocardial dysfunction is a common complication and renders septic patients at high risk of developing multiorgan failure, which is associated with high mortality. TNF-*α* has been shown to be a major factor responsible for myocardial depression during endotoxemia and cardiomyocytes are the major local source of TNF-*α*; however, so far few studies focus on the effect of propofol on cardiac function during endotoxemia or sepsis and no studies report whether propofol could inhibit LPS-induced TNF-*α* in cardiomyocytes [22, 23].

So far, many studies have reported that propofol has inhibitory effect on LPS-induced TNF-*α* generation both* in vitro* and* in vivo*. However, the molecular mechanism of how propofol exerts its inhibitory effect on TNF-*α* is still not clear. Our previous study showed that propofol can upregulate the expression of Annexin A1 in the mononuclear cells of endotoxemic rats or LPS-stimulated THP-1 cell line to reduce the release of inflammatory factors, including TNF-*α* [12]. Wu et al. found that propofol at a clinically relevant concentration can inhibit TNF-*α* biosyntheses in LPS-stimulated macrophages, and this protective effect of propofol possibly through inhibiting nuclear factor-Kappa B (NF-*κ*B) mediated increased Toll-like receptor 4 (TLR4) gene expression [24]. As for cardiomyocytes, it has been proven that gp91phox-containing NADPH oxidase activity is pivotal in LPS-induced TNF-*α* expression in the heart. This signaling pathway involves O_2_^−^ generation and activation of ERK1/2 and p38 MAPK [7]. Propofol is chemically similar to phenol-based free radical scavengers, such as endogenous antioxidant vitamin E. Each molecule of propofol can scavenge two radicals in a manner, with the phenoxyl radical reacting with a lipid peroxyl radical to form a stable nonradical adduct [25]. Based on the abovementioned background, we measured the effect of propofol on NADPH oxidase expression and NADPH oxidase activity. The concentration of propofol we chose in this study is 50 *μ*M, which is within clinically relevant concentrations and shows no toxicity to cellular viability. NADPH oxidase (nicotinamide adenine dinucleotide phosphate oxidase) is a membrane-bound enzyme complex that comprised two membrane subunits (gp91phox and p22phox, which form flavocytochrome b558) and at least four cytosolic proteins (p40phox, p47phox, p67phox, and Rac1/2, which form the cytosolic complex) [26]. As propofol accumulates in biomembranes far more readily than other antioxidants such as vitamin E, this may be one of the reasons we can see propofol significantly inhibits the expression of NADPH membrane subunit gp91phox induced by LPS. NADPH oxidase generates superoxide by transferring electrons from NADPH inside the cell across the membrane and coupling these to molecular oxygen to produce O_2_^−^ [27]. However, propofol can also scavenge free radical; therefore, it is difficult to distinguish between the effect of propofol on the scavenging of superoxide and the inhibition of NADPH oxidase. In LPS treated cardiomyocytes, we found that propofol suppresses gp91phox-NADPH expression both in mRNA and protein level and we also replicated this result in animal models. These results indicated that propofol decreases LPS-induced O_2_^−^ generation not only through scavenging them by itself but also through inhibiting the activity of gp91phox-NADPH oxidase.

It has been reported that the signaling pathway downstream of NADPH oxidase involves ERK1/2 and p38 MAPK activation [7]. We also replicated this result by using NADPH pharmacological inhibitors, DPI. In LPS treated cardiomyocytes, DPI inhibited not only NADPH oxidase activity but also the phosphorylation of ERK1/2 and p38. In the LPS plus propofol group, we got similar results, indicating that, in LPS treated cardiomyocytes, propofol suppresses the phosphorylation of p38 and ERK1/2 through its inhibitory effect on NADPH oxidase activity. Both p38 and ERK1/2 activation are required for TNF-*α* expression in LPS-stimulated cardiomyocytes. This was also demonstrated in the present study in which treatment with the p38 inhibitor SB203580 and ERK1/2 signal pathway inhibitor PD98059 both abrogated LPS-induced TNF-*α* production. So we can say the signal transduction pathway is that in LPS treated cardiomyocytes and propofol suppresses NADH oxidase expression and NADH oxidase activity to inhibit O_2_^−^ generation. Due to the decrease of O_2_^−^ generation, the phosphorylation levels of p38 and ERK1/2 are also downregulated correspondingly, ultimately leading to the declining of TNF-*α* production both in mRNA and protein levels.

Macrophages and monocytes are the predominant source of circulating TNF-*α* in response to LPS stimulation; now it is clear that cardiomyocytes are the major local source of TNF-*α* in the myocardium [28]. TNF-*α* has a central role in normal inflammatory responses and in a wide range of pathophysiologic inflammatory disorders, including sepsis. As for the pathogenesis of cardiac dysfunction in sepsis, TNF-*α* can impair contractile performance in intact animals, isolated hearts, and cardiomyocytes [4]. Furthermore, Grandel et al. have reported that LPS induced myocardial depression was completely abrogated in the presence of TNF-*α* antiserum and administration of TNF binding proteins preserved myocardial function in endotoxemic rats [29]. It has been proved that propofol could inhibit the release of TNF-*α* from macrophages and monocytes induced by LPS both* in vitro* and* in vivo *[30, 31]. Now we have demonstrated that propofol could also suppress the production of TNF-*α* in LPS treated cardiomyocytes. This may be one of the important reasons to explain why propofol could alleviate myocardial dysfunction and significantly improve survival during acute endotoxemia in mice. In the early stage of LPS-induced endotoxemia, the circulation is in a hypercoagulable state known as the pre-DIC stage, which is characterized by the adhesion and aggregation of platelet and the activation of the both extrinsic and intrinsic coagulation systems. Thrombin generation and occlusive microthrombi would cause tissue ischemia injury including heart [32]. However, we and some other researchers have previously demonstrated that propofol could reduce serum levels of platelet factor 4 (PF4) released from platelet and partially corrects the hypercoagulopathy associated with endotoxemia in rats [33, 34]. This may be another reason to explain the protective effect of propofol on cardiac system and survival in endotoxemic mice.

## 5. Conclusion

The present study demonstrated that, in LPS treated cardiomyocytes, propofol could abrogate the production of TNF-*α* through its inhibitory effect on the expression and activity of gp91phox-containing NADH oxidase. This signaling pathway involves the downregulation of O_2_^−^ generation and ERK1/2 and p38 MAPK phosphorylation. We also repeated these results in the myocardium of LPS treated mice. Due to its inhibitory effect on TNF-*α* production, we further found that propofol could alleviate cardiac depression and improve survival during endotoxemia in mice. These findings have great clinical importance for the application of propofol. In particular, when patients having heart problems endured surgical operation or systematic inflammation, propofol would be a better choice used for anesthesia or sedation, based on its anti-inflammation and antioxidant properties.

## Supplementary Material

The purity of the cardiomyocytes. Anti-troponin immunostaining was used to identify the purity of neonatal cardiomyocytes. Neonatal mouse cardiomyocytes were stained in green.

## Abbreviations

ICU: Intense care unit
TNF-*α*: Tumor necrosis factor-*α*
LPS: Lipopolysaccharides
ERK1/2: Extracellular regulated protein kinases 1/2
NADPH oxidase: Nicotinamide adenine dinucleotide phosphate-oxidase
O_2_^−^: Superoxide anion
Nox2: gp91phox
iNOS: Inducible nitric oxide synthase
IL: Interleukins
+dp/dt: Rate of contraction
CO: Cardiac output
ESPVR: End systolic pressure volume relationship
NF-*κ*B: Nuclear factor-kappa B
TLR4: Toll-like receptor 4
PF4: Platelet factor 4
DPI: Diphenyleneiodonium
MTT: 3-(4,5-Dimethylthiazol-2-yl)-2,5-diphenyltetrazolium bromide
DMSO: Dimethyl sulfoxide
i.p.: Intraperitoneal
FBS: Fetal bovine serum
PS: Penicillin-streptomycin solution
PVDF: Polyvinylidene difluoride
ELISA: Enzyme-linked immunosorbent assay
LV: Left ventricle
PBS: Phosphate-buffered saline
RT-PCR: Reverse transcription-polymerase chain reaction.
