# Supplementary material for: Propofol Inhibits Lipopolysaccharide-Induced Tumor Necrosis Factor-Alpha Expression and Myocardial Depression through Decreasing the Generation of Superoxide Anion in Cardiomyocytes
Source: Oxid Med Cell Longev. 2014 Aug 11;2014:157376. doi: 10.1155/2014/157376 (PMC4144395; doi:10.1155/2014/157376)
Supplement: Supplementary file 1 — The purity of the cardiomyocytes. Anti-troponin immunostaining was used to identify the purity of neonatal cardiomyocytes. Neonatal mouse cardiomyocytes were stained in green. [file 157376.f1.docx]

Spplementary material:

The way by which we prepared neonatal mouse cardiomyocytes is based on Dr. Qingping Feng lab’s protocol. According to anti-Troponin immunostaining, the purity of neonatal mouse cardiomyocytes is about 98%.


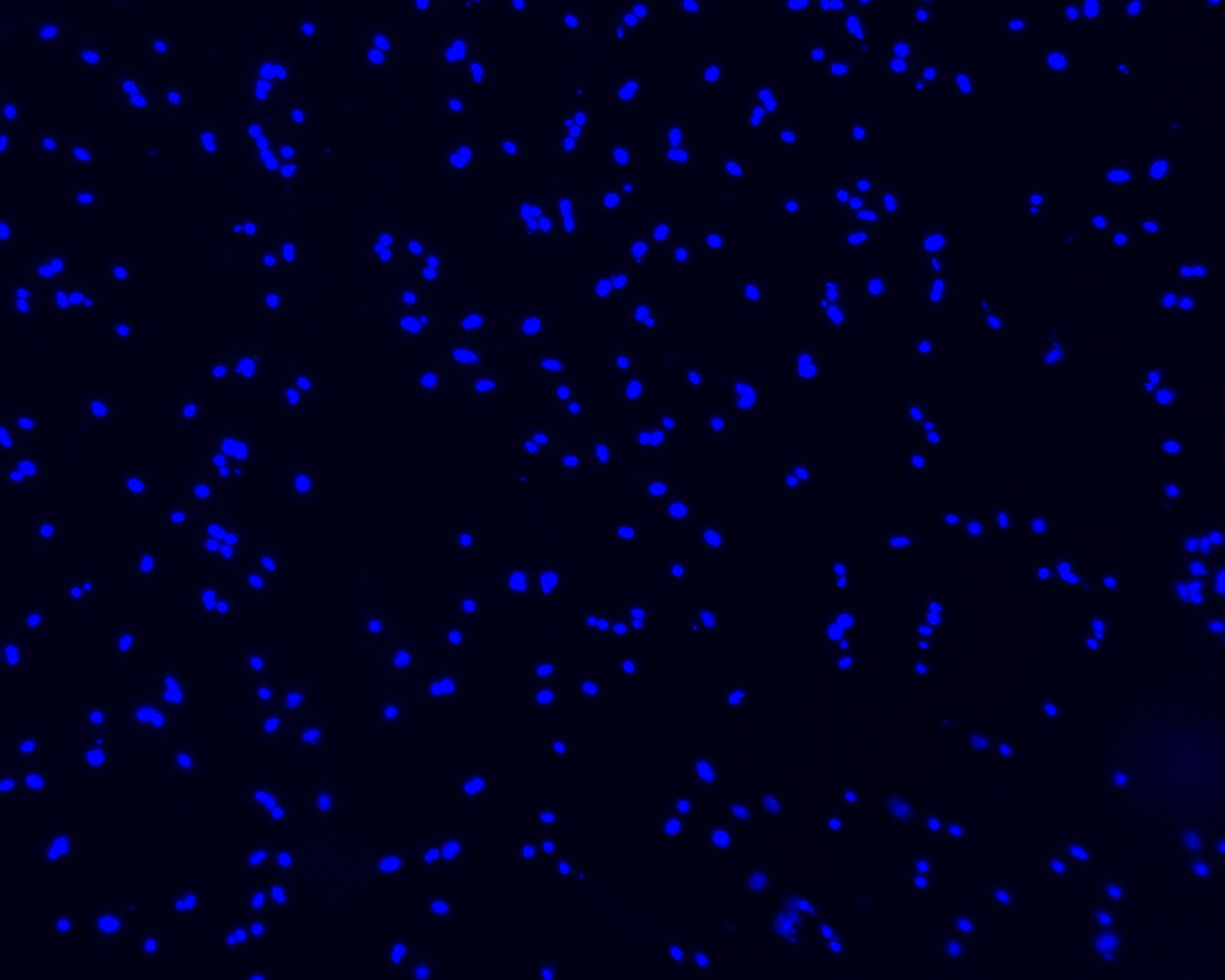

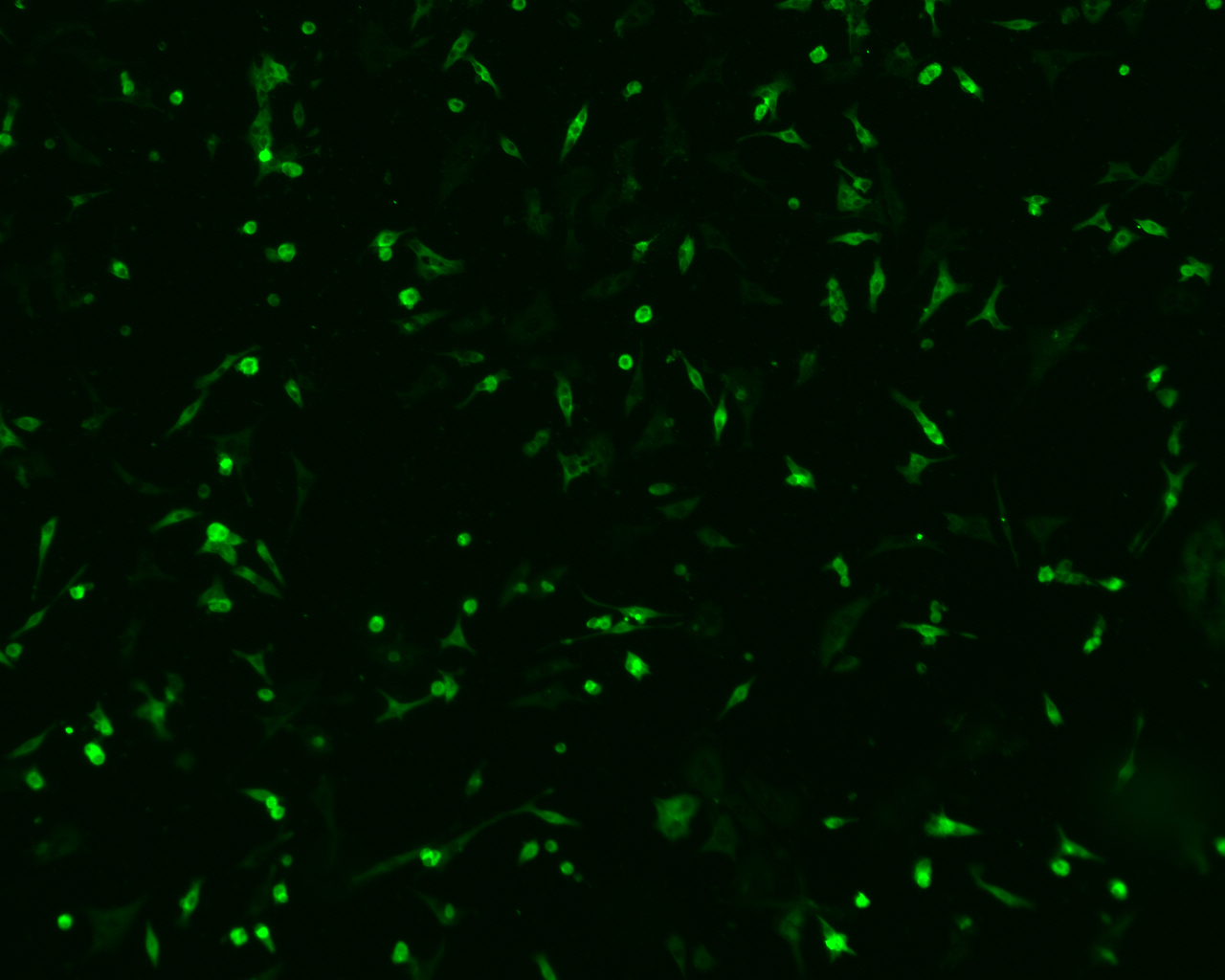

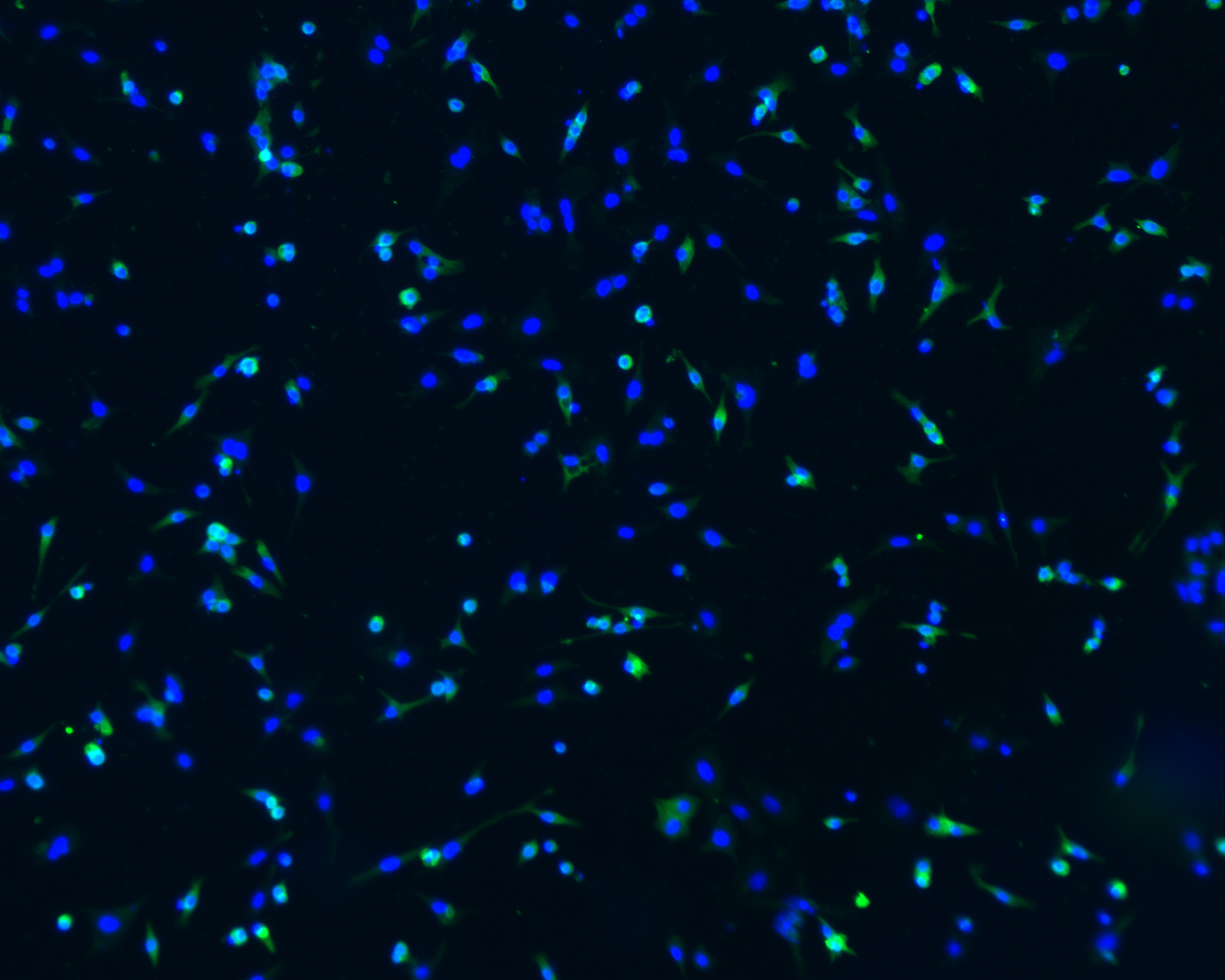


anti-Troponin

DAPI

Merge
